# Enzymatic bioconversion of blood group A to universal blood group O: a breakthrough

**DOI:** 10.1097/MS9.0000000000003971

**Published:** 2025-09-29

**Authors:** Mashal Hafeez, Zain Mustafa, Noor Un Nisa Irshad, Sakan Binte Imran

**Affiliations:** a Department of Medicine, Bakhtawar Amin Medical & Dental College, Multan, Pakistan; b Department of Medicine, Jinnah Sindh Medical University, Karachi, Pakistan; c Department of Medicine, Sir Salimullah Medical College, Dhaka, Bangladesh

*Dear Editor,*

WHO reports that approximately 120 million units of blood donations are collected globally annually^[1]^. However, the global demand exceeds this amount, and blood banks need to be supplemented regularly with donations so that blood is readily available to patients requiring transfusions. This article is in line with the TITAN Guidelines on the need for transparency in AI use in healthcare^[2]^.

Currently, 30 discrete blood groups are known^[3]^, and the most clinically significant amongst them is the ABO blood group system. This system consists of A, B, AB, and O blood groups. O type is the universal donor as it lacks any glycoprotein moieties, and therefore has no A or B antigens. The base structure of this system is a fucosyl galactose moiety bound to *N*-acetylglucosamine, which represents the H-antigen of O blood group. The key difference between O- and A-blood groups is the terminal monosaccharide, α-1,3-linked *N*-acetyl galactosamine (GalNAc) attached to H-antigen present in A-type blood^[4]^, which raises the possibility of conversion of type A blood to universal type O blood by enzymatic cleavage.

Successful interconversion of blood group types could potentially pave the way for safer and effective treatment opportunities, such as faster access to organ/tissue transplants, decreased chances of graft rejection, reduced risk of transfusion-transmissible diseases, or adverse transfusion outcomes, etc.

Certain glycoside hydrolases are capable of removing the A antigen in blood group A to form O blood. For example, *N*-acetyl galactosamine is the only enzyme belonging to the GH109 family that can form type O red blood cells (RBCs) from type A RBCs^[5]^.

A new study has also discovered two enzymes obtained from *Flavonifractor plautii, N*-acetyl galactosamine deacetylase (*Fp*GalNAcDeAc) and galactosaminidase (*Fp*GalNase), which are capable of removing A antigens from type A blood and converting it into universal type O blood^[6]^.

This theory was put to the test in an experiment in which they extracted these two enzymes and incubated them with type A RBCs for 5 minutes^[5]^. Ninety-nine percent of A-type RBCs were successfully converted to O-type RBCs. The results also showed improved catalytic efficiency at higher enzyme concentrations and with covalently-linked fusion proteins (FpGalNAcDeAc–FpGalNase and FpGalNase–FpGalNAcDeAc).

Furthermore, these two enzymes were also utilized to convert type A kidneys to universal type O kidneys^[7]^. This feat was achieved by using two machine perfusion techniques, normothermic machine perfusion (NMP) and hypothermic machine perfusion to perfuse the organ ex vivo and convert it into a nonimmunogenic state. The results indicated an almost 80% conversion of A antigens to H antigens in under 2 hours, while renal parameters remained stable for both strategies. However, this conversion rate is still less than the 97% conversion in 4 hours achieved during enzymatic perfusion treatment of lungs. In that experiment, the *ex vivo* lung perfusion technique was used to perfuse the donor lung outside the body and effectively convert 99% of type A RBCs into universal type O RBCs^[8]^.

Moreover, previously in 2023, a research study had reported the successful bioconversion of group B kidneys to group O kidneys by the enzyme α-galactosidase, although the type of machine perfusion was limited to NMP only^[9]^.

The enzymatic bioconversion of blood group A to universal group O represents a significant advance in transfusion and transplant medicine. Emerging evidence demonstrates that specific glycoside hydrolases, such as those derived from *F. plautii*, can effectively remove A antigens from both RBCs and organ tissues under *ex vivo* conditions. While these findings are promising, further research is needed to evaluate the safety, efficacy, and feasibility of such approaches in clinical settings. If validated in larger studies, this technology could expand the donor pool, reduce immunological barriers, and improve equity in access to blood and organ resources.

## Data Availability

Not applicable.
